# Rationalizing the Role of Monosodium Glutamate in the Protein Aggregation Through Biophysical Approaches: Potential Impact on Neurodegeneration

**DOI:** 10.3389/fnins.2021.636454

**Published:** 2021-03-04

**Authors:** Ishfaq Ahmad Ahanger, Sania Bashir, Zahoor Ahmad Parray, Mohamed F. Alajmi, Afzal Hussain, Faizan Ahmad, Md. Imtaiyaz Hassan, Asimul Islam, Anurag Sharma

**Affiliations:** ^1^Department of Chemistry, Biochemistry and Forensic Science, Amity School of Applied Sciences, Amity University Haryana, Gurgaon, India; ^2^Centre for Interdisciplinary Research in Basic Sciences, Jamia Millia Islamia, New Delhi, India; ^3^Department of Pharmacognosy College of Pharmacy, King Saud University, Riyadh, Saudi Arabia

**Keywords:** monosodium glutamate, protein aggregation, nucleation-dependent polymerization, isothermal titration calorimetry measurement, neurodegeneration

## Abstract

Monosodium glutamate (MSG) is the world’s most extensively used food additive and is generally recognized as safe according to the FDA. However, it is well reported that MSG is associated with a number of neurological diseases, and in turn, neurological diseases are associated with protein aggregation. This study rationalized the role of MSG in protein aggregation using different biophysical techniques such as absorption, far-UV CD, DLS, and ITC. Kinetic measurements revealed that MSG causes significant enhancement of aggregation of BSA through a nucleation-dependent polymerization mechanism. Also, CTAB-BSA aggregation is enhanced by MSG significantly. MSG-induced BSA aggregation also exhibits the formation of irreversible aggregates, temperature dependence, non-Arrhenius behavior, and enhancement of hydrodynamic diameter. From the isothermal titration calorimetry measurement, the significant endothermic heat of the interaction of BSA-MSG indicates that protein aggregation may be due to the coupling of MSG with the protein. The determined enthalpy change (Δ*H*) is largely positive, also suggesting an endothermic nature, whereas entropy change (Δ*S*) is positive and Gibbs free energy change (Δ*G*) is largely negative, suggesting the spontaneous nature of the interaction. Furthermore, even a low concentration of MSG is involved in the unfolding of the secondary structure of protein with the disappearance of original peaks and the formation of a unique peak in the far-UV CD, which is an attention-grabbing observation. This is the first investigation which links the dietary MSG with protein aggregation and thus will be very instrumental in understanding the mechanism of various MSG-related human physiological as well as neurological diseases.

## GRAPHICAL ABSTRACT 1

Kinetic measurements indicating that monosodium glutamate causes significant enhancement of aggregation of protein through nucleation-dependent polymerization mechanism. Even low concentration of MSG is involved in the unfolding of secondary structure of protein with the disappearance of original peaks (208 and 222 nm) and formation of unique peak (226 nm) in the far-UV CD.

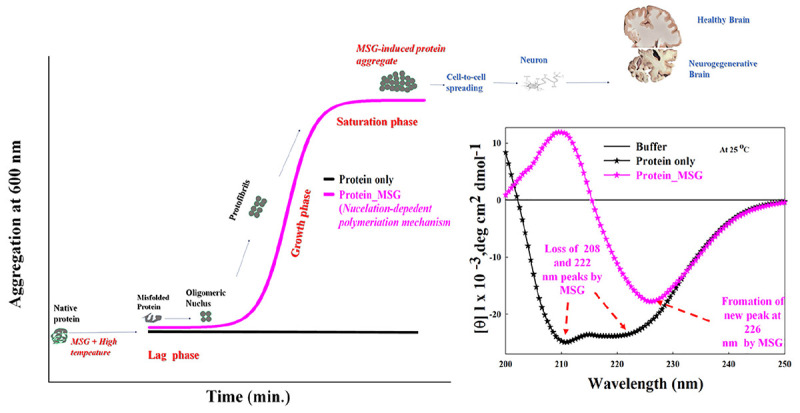

## Introduction

**FIGURE 1:**
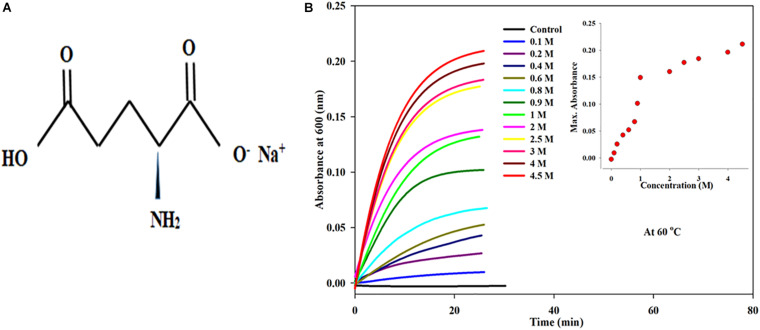
**(A)** Chemical structure of monosodium glutamate (MS). **(B)** Kinetic measurements of BSA in presence increasing concentrations of monosodium glutamate (0.1 to 4.5 Molar) at 60°C temperature, 25 mM phosphate buffer, pH. 7.0 and 600 nm. Inset of Figure 1 inset shows the influence of concentration on (*A*_*max*_) maximum absorbance of MSG-induced BSA aggregation at 60°C temperature.

Monosodium glutamate (MSG), chemical formula C_5_H_8_NO_4_Na, is the sodium salt of glutamic acid and is naturally found in cheese, tomatoes, mushroom, and grapes (Jinap and Hajeb, 2010; Figure 1A). MSG is also known as Ajinomoto or Chinese salt and sodium 2-aminopentanedioate (IUPAC name). Structurally, it is composed of 12% sodium cations (Na^+^), 78% glutamate anions (C_5_H_8_NO_4_^–^), and 10% water. Sodium cations and glutamate anions in MSG are held by an ionic bond. It acts as a powerful flavor enhancer due to its unique fifth taste called umami or savory or meaty taste, so MSG is one of the world’s most extensively used food additives and is generally recognized as safe according to the Joint FAO/WHO Expert Committee on Food Additives (JECFA), the Food and Drug Administration (FDA), and the European Food Safety Association (EFSA) (Yadav, 2010). However, there are numerous reports regarding the harmful effects of food containing excessive amounts of MSG and how it causes headache, migraine, tingling or burning in the face, sweating, facial pressure or tightness, numbness, flushing in the neck and other areas, rapid fluttering heartbeats (heart palpitations), chest pain, nausea, and weakness, according to the International Classification of Headache Disorders (ICHD), third edition. Such physiological responses are referred to as MSG symptom complex or Chinese restaurant syndrome (Yang et al., 1997).

Structurally, the core component of MSG is glutamate, which is a negatively charged, polar, and nonessential amino acid (i.e., can be synthesized by the human body and so is not essential to human diet). It has been suggested that glutamate bound to other amino acids in proteins does not show any taste; however, glutamate can show taste when it is unbound or in free form (Löliger, 2000). In the free or unbound form, glutamate stimulates the glutamate receptors (TAS1R1, TAS1R, mGluR4, and mGluR1), which are located in the taste buds and are responsible for inducing the flavor profile called umami (Conn and Pin, 1997). There are various studies which suggested that thymocytes, lymphocytes, and thymic stromal cells also possess the receptors for glutamate (Pavlovic et al., 2006). Further, it is reported that MSG comprises two isomers, i.e., L-glutamate and D-glutamate enantiomers, but only L-glutamate enantiomer is responsible for enhancing the flavor (Okoye et al., 2016). Processed MSG comprises 99.6% L-glutamate form which is a greater percentage of L-glutamate than that observed in the free glutamate ions of naturally occurring foods (Halpern, 2000). When ingestion of MSG occurs orally in humans, it splits into glutamate and sodium ions in the digestive tract. In the gut and intestine, glutamate is absorbed by active transport. The absorbed glutamate is carried from the intestine to the lumen across the apical membrane with the help of transporters like EAAC-1 (excitatory amino acid transporter) and NaDC-1 (sodium carboxylate transporter) (Kanai and Hediger, 2003). These transporters transport glutamate into the blood stream and circulate throughout the body. In the intestinal enterocyte, catabolism of glutamate initiates in the cytosol and mitochondria through the phenomenon of transamination in the presence of aspartate aminotransferase, alanine aminotransferase, branched-chain aminotransferase, and glutamate dehydrogenase (GDH) enzymes, resulting in the formation of end product α-ketoglutarate. α-Ketoglutarate enters into a TCA cycle with the release of carbon dioxide (Windmueller and Spaeth, 1976). Moreover, it has been reported that consumption of MSG results in the formation of nine urinary metabolites, which include glutamate, alpha-ketoglutrate, malonate, citrate, 5-aminovalerate, 5-hydroxymethyl-4-methyluracil, dimethylamine methylamine, and beta-hydroxyisovalerate. Thus, the elimination of MSG in the form of these urinary metabolites occurs through the kidneys (Nahok, 2019).

Glutamate is also an excitatory neurotransmitter present in the central nervous system (brain and spinal cord) and by means of the (vGlut) vesicular glutamate transporter family; glutamate is transported into the synaptic vesicle. It is well reported that the excitotoxic behavior of glutamate results in neurodegenerative diseases (Terlisner, 2009). Likewise, MSG has been observed in connection with neurotoxicity, which refers to damage of the central or peripheral nervous system. The damage of neurons by the overstimulation or overactivation of glutamate receptors by excitatory neurotransmitter glutamate may lead to the death of neurons by an act of excitotoxins; the process is called excitotoxicity (Choi, 1988). During excitotoxicity, there is excessive discharge of glutamate from glutamatergic nerve terminals, which leads to the overactivation of glutamate receptors (NMDA receptor and AMPA receptor), resulting in an increase in the level of intracellular calcium ions (Ca^2+^). This increase in the level of calcium ions causes the activation of enzymes like endonucleases, phospholipases, and proteases (such as calpain), which are responsible for the damage of cytoskeleton, membrane, DNA, and especially the structure of neurons. Even the elevation of Ca^2+^ is involved in mitochondrial stress, causing extreme oxidative phosphorylation and production of reactive oxygen species (ROS) by triggering nitric oxide synthase, eventually resulting in cell death (Mehta, 2013). It has been observed that a rise in glutamate beyond the optimum concentration of about 1 mM in the synaptic cleft leads to a degeneration of neurons (Clements et al., 1992).

There are numerous reports which suggests that excessive use of MSG in the food is linked with FM (fibromyalgia) (Smith et al., 2001), hyperaggregation of platelets in rats (H.M.Youssef et al., 2010), atrial fibrilization (Shi et al., 2011), hypatoxicity (Egbuone, 2009), nephrotoxicity of endocrine disruptors, hyperglycemia consequently with diabetes mellitus (Ogbuagu, 2019), and overweight (Zanfirescu, 2019). Furthermore, in rats it is revealed that the platelet count, bleeding, and coagulation time increase by the dietary ingestion of MSG (Ajibola, 2012). There are numerous recent reports which highlights that the orally taken MSG increases the free radical formation, escorts DNA damage and triggers the apoptotic signaling cascade, which subsequently onset repair mechanism in stem cells and leads to neural cell necrosis (Mathew, 2019). Thus, it is a promoter of neurodegenerative diseases such Alzheimer’s diseases, Parkinson’s disease, Huntington’s disease, amyotrophic lateral sclerosis, and multiple sclerosis (Fuchsberger, 2019).

Generally, it is a well-known fact that neurological diseases are related with the phenomenon of misfolding and protein aggregation, and this phenomenon may be defined as the cellular event by which proteins are unable to fold into functional form resulting in misfolded proteins, which in turn polymerizes into aggregates, thereby amalgamating together intracellularly as well as extracellularly, forming a structurally and functionally abnormal assembly and triggering a wide variety of pathological and neurological diseases (Chiti and Dobson, 2006; Ahanger et al., 2021). Protein aggregate is the protein in the nonnative state with at least twice size of native protein, i.e., 100 to 1000 nm. It shows fewer properties like reduced activity or no biological activity (Dobson, 2003; Chiti and Dobson, 2006). Protein misfolding and aggregation are currently the primary source of apprehension in biology and medicine due to their association with various debilitating human neurodegenerative proteinopathies, including Alzheimer’s, Parkinson’s disease, amyotrophic lateral sclerosis, and Huntington’s disease. The mutations, errors during protein synthesis, aging, environmental stress, and formation of ROS (reactive oxygen species) (Tyedmers et al., 2010) are some possible causes that can result in the formation of protein misfolding and aggregation. Furthermore, pH (Katayama et al., 2006), temperature, protein concentration (Ghosh et al., 2018), additives (Hamada et al., 2009), and viscosity (Barnett et al., 2015) are some factors which can influence the protein aggregation. Despite having numerous aforementioned reports regarding the association of MSG with various neurological disorders, still there are no reports available about the role of MSG in protein misfolding and aggregation, or the role of MSG has not been explored yet in protein misfolding and aggregation. Thus, there is a need for vigorous investigations regarding the role of MSG in protein misfolding and aggregation.

Investigations of various aspects such as human complications and proteinopathies including neurodegenerative diseases can be executed and studied by promoting and inhibiting protein aggregation in *in vitro* experiments (Bhattacharyya and Das, 1999). Bovine serum albumin (BSA) has been often used as the best model protein for studying *in vitro* protein misfolding and aggregation by virtue of its strong ligand-binding capacity; being a carrier for vitamins, fatty acids, hormones, iron, drugs, and trace minerals; having a structural similarity with human serum albumin; and osmotic pressure and pH of blood being also controlled through serum albumin in circulation (Carter and Ho, 1994). Therefore, the fundamental purpose of this study was to induce the aggregation in bovine serum albumin using high temperature in the presence of MSG and to unfold the effective role of MSG in delaying or increasing protein aggregation. This study will provide some explanation for various MSG-induced neurodegenerative and physiological complications. Different spectroscopic techniques such as absorption spectroscopy (Moosavi-Movahedi et al., 1996), fluorescence spectroscopy (De et al., 2005), circular dichroism (Gelamo and Tabak, 2000), dynamic light scattering (Kelley and McClements, 2003), and isothermal titration calorimetry (Parray et al., 2019) were used for this purpose.

## Materials and Methods

### Materials

Bovine serum albumin (BSA, UniProtKB-A0A140T897_BOVIN: A0A140T897) used was lyophilized, which was obtained from Sigma-Aldrich. Dietary MSG (MSG, CAS number: 142-47-2) and cetrimonium bromide (CTAB) were obtained from Sigma-Aldrich. Merck (India) provided chemicals like monobasic, i.e., (NaH_2_PO_4_) sodium dihydrogen phosphate, and dibasic, i.e., disodium hydrogen phosphate (Na_2_HPO_4_) and sodium dihydrogen phosphate (NaH_2_PO_4_), which were used to prepare the solution of phosphate buffer. The phosphate buffer system in which all experiments were done after calculating its strength was kept 25 millimolars with 7.0 pH, and a suitable amount of both monobasic and dibasic was added in Milli-Q water which was obtained from the Millipore system. All the chemicals received were directly used with no more extra purification. The stock of BSA protein solution was formulated with the concentration of 15 milligrams per milliliter. By using the Varian Cary 100 Bio double-beam spectrophotometer, the absorbance of protein aliquot from the stock at the wavelength of 278 nm and 44,000 molar extinction coefficient as well as the concentration of protein stock was measured. A stock of 5 molars of MSG and 25 millimolars of surfactant CTAB was also formulated in the phosphate buffer at pH 7.0. The pH’s of all the above solutions were measured with the help of Toshcon Digital pH Meter CL-54+.

### Methods

#### Kinetic Measurements

The kinetic aggregation of BSA protein solution was carried out by monitoring its turbidity in the presence of MSG through time course measurement using the Jasco V-660 UV-vis spectrophotometer (JASCO Corporation 2967-5, Ishikawa-machi, Hachioji-shi, Tokyo, Japan) at the wavelength of 600 nm. The spectrophotometer was coupled with setup of a Peltier temperature regulator (ETCS61) to control the temperature. The study of aggregation kinetics of BSA was carried out in the presence of varying concentrations of MSG ranging from 0.1 M to 4.5 M and also at different temperatures ranging from 60, 70, 75,78, 81, 82, 84 to 85°C. To avert and tackle the hindrance created by absorbance of chromophoric groups when the light interacts with protein, the turbidity measurements of protein solution are generally executed above the higher wavelength 400 nm. It has been suggested that aggregates of proteins are characterized with high optical density and high turbidity. The curve fitting was done to data acquired after the experiments of kinetic measurements by applying a four-parameter logistic curve in the above mathematical statement (Sharma et al., 2010a,b; Ahanger et al., 2021):

(1) $$y=y_{0}+\frac{a}{1+\text{exp}\,\left(-\left(t-t\,1/2\right)/b\right)}$$

where *y* is the absorbance at any time *t*, *y*_*o*_ is the initial absorbance value, *a* is the maximum absorbance, t_1/2_ is the time at which absorbance is half of its maximum, *b* is 1/*k*_*app*_ (reciprocal apparent rate constant), apparent rate constant *k*_*app*_ is 1/*b*, and lag time, *t*_*lag*_ = *t*_1/2_−2*b*.

##### Kinetic measurements of BSA in the presence of fixed concentration of monosodium glutamate at various temperatures

The effect of temperature on kinetic measurements of BSA in the presence of fixed concentrations of MSG was also checked. The rate constants, i.e., the rate constant coupled with nucleation phase (1/tlag) and the apparent rate constant coupled with growth phase (*k*_*app*_) calculated from this experiment, were applied to evaluate the activation energy of aggregation through using equations (Sharma et al., 2010a,b) (2) and (Sharma et al., 2010a,b) (3):

(2) $$\left(\frac{(\Delta ln\left(1/tlag\right)}{\Delta \,T}\right)=\frac{-E\,a,n\,u\,c}{R\,T^{2}}$$

(3) $$\left(\frac{(\Delta ln\left(k\right)}{\Delta \,T}\right)=\frac{-E\,a,e\,l\,o\,n\,g}{R\,T^{2}}$$

#### Structural Measurements

##### Dynamic light scattering measurements

To measure the size distribution profile particle and the values of hydrodynamic radius (*R*_*h*_) in the solution (in which agglomerates of BSA in the presence of MSG -induced BSA aggregates were removed by centrifugation), dynamic light scattering (DLS) was performed using RiNA Laser Spectroscatter, Model-201 (RiNA GmbH Berlin, Germany), at 25 ± 0.1°C. The protein solutions were filtered through Millipore syringe filters with the size of 0.22 μm. Polystyrene was applied to make the disposable cell with the scattering angle of 90°. The measuring light scattering intensity of fluctuations is the basic principle of analysis of particle size distribution profile in the protein solution. Therefore, the size distribution profile of BSA-MSG was acquired through determining the time-dependent fluctuations of scattered light which are caused by particle proteins moving in the solution under the influence of Brownian motion. From these fluctuations, diffusion coefficients of BSA-MSG can be determined mathematical formula (Parray et al., 2019) (4).

(4) $$D=\frac{\text{kT}}{6\,\pi \,\eta \,\text{Rh}}$$

where *D* is the diffusion, k is Boltzmann’s constant, *T* is the temperature, η is the shear viscosity of solvent, and *R*_*h*_ is the hydrodynamic radius of the particle. The data was analyzed by using the PMgr version 3.01 software.

##### Circular dichroism spectroscopy measurements

Far UV-CD analysis of a protein solution with a concentration of 5 micromolars, in a cuvette with path length 0.1 cm recorded with J-1500 Circular Dichroism Jasco Spectropolarimeter (JASCO International Co. Ltd, Tokyo, Japan). The machine is connected with a mini-Jasco circulatory water bath (MCB-100) and a very effective nitrogen purge system. The CD experiment was performed at room temperature with wavelength 200 nm. To calibrate the machine regularly, D-10 camphorsulfonic acid (CSA) was used. The data obtained from the CD are averages of three scans for a sample, and this acquired data in t units of millidegree was converted into mean residual ellipticity.

##### Isothermal titration calorimetry studies

Isothermal titration calorimetry (ITC) is a biophysical method applied to evaluate the binding interaction and thermodynamic parameters of protein with ligand in the solution by calculating the heat that is either liberated or consumed. A VP ITC Calorimeter (MicroCal, 22 Industrial Drive East, Northampton, MA 01060, United States) instrument was used for ITC measurement, at 25°C in 25 mM phosphate buffer (pH 7.0), in which the calorimeter cell was injected with a fixed concentration of 30 μM protein solution. During the ITC measurement, the ligands with the concentration of 600 μM MSG were titrated against the cell having 30 μM BSA. Each ligand solution was loaded with 10-microliter aliquots in each 260-second step through the syringe, and each ligand was loaded into control, i.e., phosphate buffer. The normalization of data was done with the results of titration of respective ligands, and MicroCal Origin ITC software was used for fitting the data to produce the profile of heat change. From the measured heat changes, the stoichiometry (N), binding enthalpy (Δ*H*), and association constant (*K*_*a*_) were calculated on binding of MSG with BSA. Gibbs-free energy changes (Δ*G*) were also elevated from the calculated heat changes by means of the following equation (Parray et al., 2019; Ahanger et al., 2021):

(5) Δ⁢G=-R⁢T⁢l⁢n⁢K⁢a=Δ⁢H-T⁢Δ⁢S

where *R* is the gas constant and *T* is the absolute temperature.

## Results

### Kinetic Measurements of BSA in the Presence of Increasing Concentrations of Monosodium Glutamate at 60°C Temperature

By means of time course measurement using the UV-visible spectrophotometer, the aggregation kinetics of bovine serum albumin in the presence of dietary MSG was done by assessing the turbidity of the protein solution. The aggregation kinetics was performed on a protein solution with a fixed volume of 5 μM by titrating with different concentrations of MSG starting from 0.1 molar to 4.5 molar. The experiment was done at a wavelength of 600 nm and at 60°C in 25 millimolar phosphate buffer at pH 7.0. It is clear from Figure 1B that dietary MSG increases the BSA aggregation denoted as a function of time with the enhancement in absorbance at the wavelength of 600 nm. There was no aggregation observed in 5 μM BSA (in buffer as control), but as the concentration of MSG was varied from 0.1 to 4.5 M, the tendency of aggregation also increased with the increase in absorbance from 0.00289 to 0.2110. This MSG-induced BSA aggregation is represented by a sigmoidal curve, marked by the absence of a nucleation phase with insignificant absorbance, steeply followed by a growth phase which was stabilized by a saturation phase.

### Kinetic Measurements of BSA in the Presence of Increasing Concentrations of **Monosodium Glutamate a**t 70°C Temperature

**FIGURE 2:**
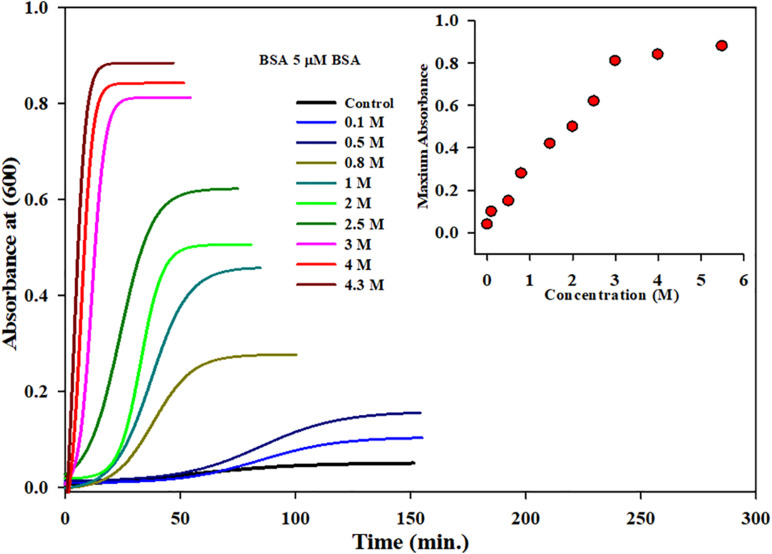
Kinetic measurements of BSA in presence increasing concentrations of monosodium glutamate (0.1 to 4.5 Molar) at 70°C temperature, 25 mM phosphate buffer, pH. 7.0 and 600 nm. Inset of Figure 2 inset shows the influence of concentration on (*A*_*max*_) maximum absorbance of MSG-induced BSA aggregation at 70°C temperature.

All the parameters were the same in the aggregation kinetics as the 5-μM protein concentration was taken with varying concentrations of MSG starting from 0.1 molar to 4.3 molar. The experiment was done at wavelength of 600 nm at 70°C of temperature in 25 millimolar phosphate buffer with pH 7.0. It is clear from Figure 2 that dietary MSG increases the BSA aggregation denoted as a function of time with the enhancement in absorbance at the wavelength of 600 nm. There was no aggregation observed in 5 μM alone (taken as control), but as the concentration of MSG was increased from 0.1 to 4.3 M, there was a significant increase in the absorbance observed from 0.04 to 0.88 nm. Furthermore, an increase in the 10-degree temperature form, from 60 to 70°C, MSG was involved in the induction of a well-established lag phase in the process of aggregation which was not observed at 60°C temperature. This MSG-induced BSA aggregation is represented by a sigmoidal curve, marked by the well-established lag or nucleation phase, steeply followed by the growth phase which was stabilized by the saturation phase.

**FIGURE 3:**
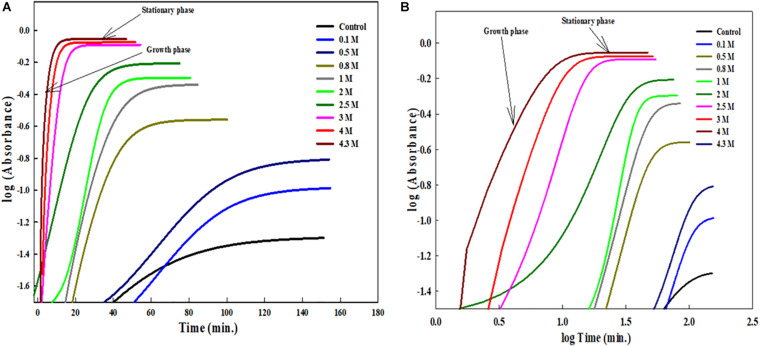
**(A)** Shows the log-lin plot of absorbance versus time and **(B)** shows the log-log plot of absorbance versus time for BSA aggregation at 70°C temperatures in the presence of increasing concentration of MSG.

The insets of Figures 1B, 2 represent the plot of maximum absorbance versus concentration (M) which suggests that MSG-induced aggregation of BSA is nonlinearly dependent on the concentration. Through applying the log–lin plot of log absorbance versus time and the log–log plot of log absorbance versus log time, the mechanism of MSG-induced protein aggregation was described as depicted in Figures 3A,B.

### Kinetic Measurements of BSA in the Presence of Monosodium Glutamate at Various Temperatures

**FIGURE 4:**
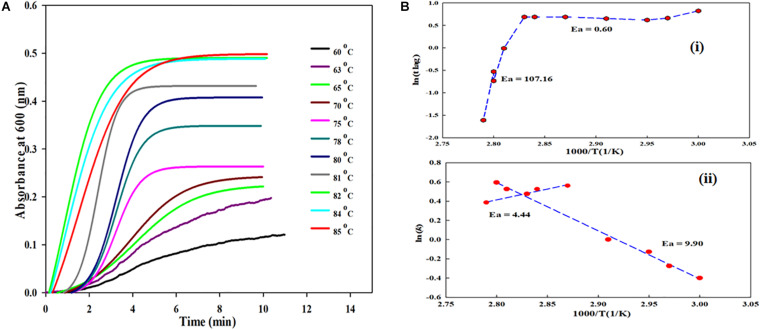
**(A)** Kinetic measurements of BSA in presence fixed concentrations of monosodium glutamate at various temperatures (60°C, 63°C, 65°C, 70°C, 75°C, 78°C, 80°C, 81°C, 82°C, 84°C, and 85°C). **(B) (i)** shows plot between lnt*_*lag*_* versus the reciprocal of temperature for MSG-induced aggregation of BSA and **(B) (ii)** shows plot between ln*(k)* versus the reciprocal of temperature for MSG-induced aggregation.

In this experiment, the aggregation kinetics of 5 μM BSA were carried out in the presence of a fixed concentration (1 molar) of MSG at various temperatures (60°C, 63°C, 65°C, 70°C, 75°C, 78°C, 80°C, 81°C, 82°C, 84°C, and 85°C) depicted in Figure 4A. It is certain from this figure that, as the temperature increases from 60°C to 85°C, the MSG-induced aggregation of BSA is significantly enhanced, with the increase in the absorbance from 0.120 to 0.4 94. The turbidity of the solution was noticed to be lesser at 60°C; however, there is significant rise in the turbidity of the solution after 60°C temperature.

The plots between lnt*_*lag*_* and ln(*k*) versus the reciprocal of temperature for MSG-induced aggregation of BSA are shown in Figures 4Bi,ii. The Figure 4Bi plot illustrates the nonlinear relationship between the reciprocal of temperature and ln t*_*lag*_*, and with the drop in temperature, t*_*lag*_* gets prolonged. Further, the activation energies (Ea) of kinetics aggregation of BSA in the presence of MSG were calculated from the plots of lnt*lag* vs. 1000/T and ln(*k*) vs. 1000/T which largely show a nonlinear behavior. The nonlinear fit curve made up of two linear segments and the complete curve was fitted with two linear equations, which in turn gives two activation energies, i.e., 60 to 80°C which is 0.60 kcal/mol and 80 to 85°C which is 107.16 kcal/mol. Also, the activation energy from 60°C to 80°C is 9.90 kcal/mol and that from 80°C to 85°C is 4.44 kcal/mol, calculated from the linear fit of the plot of ln(*k*) vs. 1000/T. The activation energy calculated from the plot of ln tlag vs. 1000/T is accompanied with the nucleation phase of aggregation whereas the activation energy calculated from the plot of ln(*k*) vs. 1000/T is accompanied with the growth phase of aggregation. Thus, the temperature is an important factor on which MSG-induced BSA aggregation is dependent.

### Influence of Monosodium Glutamate on the Kinetics of CTAB-Induced Aggregation of BSA

**FIGURE 5:**
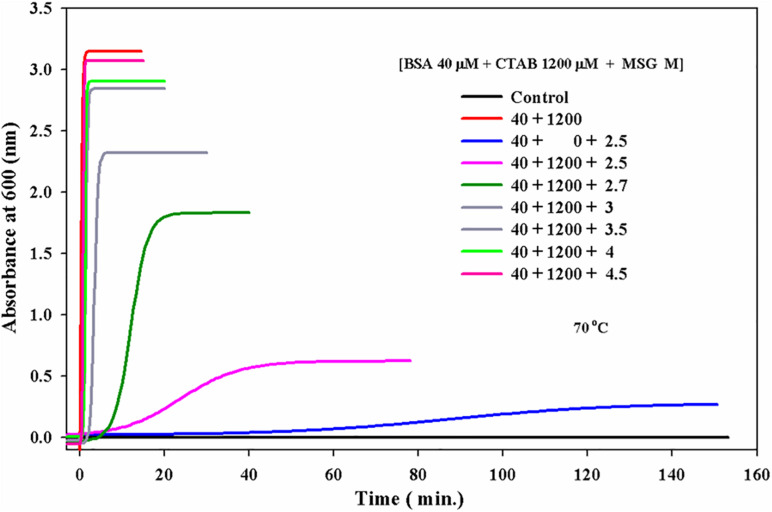
Effect of monosodium glutamate on the kinetics of CTAB-induced aggregation of BSA at 70°C temperature 25 mM phosphate buffer, pH 7.0 and wavelength of 600 nm.

The aggregation kinetics was carried out in the presence of MSG and surfactant cetyltrimethylammonium bromide (CTAB) at 70°C shown in Figure 5. In this experiment, the ratio of BSA and CTAB was kept fixed at 1:30, i.e., the concentration of the protein solution was maintained at 40 micromolars of BSA and the concentration of CTAB was maintained at 1200 micromolars and only the concentration of MSG was varied from 2.5 to 4.5 M. Figure 5 indicates that BSA at 40 μM does not show the aggregation with the absorbance of 0.00076. Once 1200 μM CTAB was added to the reaction mixture, there was a marked increase in the aggregation with the absorbance of 3.15.

After that, this CTAB-induced aggregation was carried out in the presence of increasing concentration of MSG from 2.5 to 4.5 molars. From Figure 5, it is clear that with the increase in concentration of MSG from 2.5 to 4.5, there is further increase in the aggregation of BSA in the presence of CTAB. CTAB-induced BSA aggregation in the presence of increasing concentration of MSG from 2.5 to 4.5 molars with the absorbance of 3.08 was found very close to CTAB-induced BSA aggregation (i.e., absence of MSG) with the absorbance of 3.1. This indicates that MSG is not involved in the inhibition of aggregation of BSA even in the presence of CTAB. Supplementary Figure 1 shows that both MSG and CTAB act as the homologous seeds in the nucleation phase of BSA aggregation.

### Influence of Monosodium Glutamate on Reversibility of BSA

In this experiment, a UV-visible spectrum (in the range of 230 to 340 nm wavelength) of 5 μM of BSA was taken at the temperature of 25°C in the absence of MSG. At 280 nm, there was no scattering observed and the absorbance of this sample was 0.308. However, when the UV-Visible spectra were taken in the presence of MSG, at 280 nm, there was marked scattering observed and the absorbance of this sample was 0.727. BSA in the presence of MSG was subjected to thermal aggregation at a temperature of 80°C and subsequently the UV-Visible spectra were taken to this aggregated sample. The aggregated sample was extremely turbid, and there was no peak observed at all. Moreover, enormous scattering is observed and the absorbance of this sample at 208 nm is 1.188.

**FIGURE 6:**
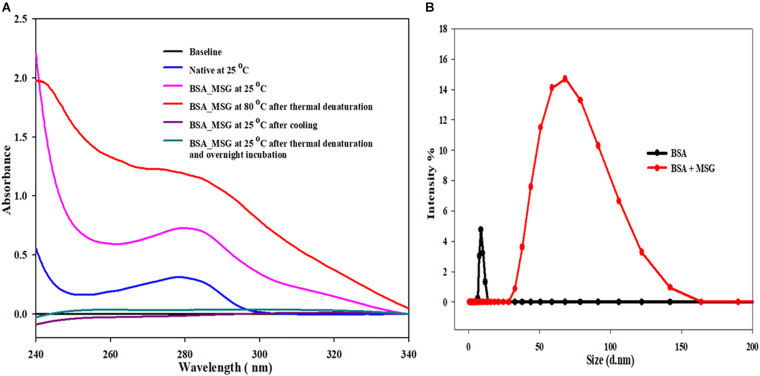
**(A)** Determination of reversibility of BSA in the presence of monosodium glutamate. **(B)** Influence of MSG on the measurement of the hydrodynamic size of bovine serum albumin.

After the thermal aggregation, two UV-visible spectra of this aggregated sample were taken, once on cooling after half an hour at 25°C and another after overnight incubation at 25°C. In both the cases, all the peaks were removed and the spectra of thermally aggregated BSA in the presence of MSG were completely flattened. From this experiment, as shown in Figure 6A, it is clear that the BSA structure is perturbed greatly in the presence of MSG and at high temperature there is the formation of aggregates of BSA induced by MSG and these aggregates are completely irreversible in nature.

### Effect of Monosodium Glutamate on the Measurement of the Hydrodynamic Size of BSA

Determining the size of a BSA protein in the absence or in the presence of MSG at room temperature, DLS measurements were performed. Figure 6B shows the results of DLS measurement, in which there is marked increase in the hydrodynamic diameter of BSA in the presence of MSG from 8.3 to 68.33 nm. From the results, it is clear that addition of MSG causes the aggregation of BSA with the increase in size.

### Effect of Monosodium Glutamate on the Secondary Structure of Bovine Serum Albumin by Performing Far-UV CD Measurements

**FIGURE 7:**
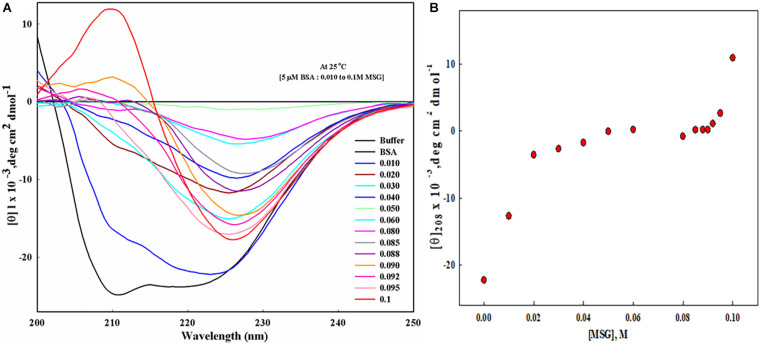
**(A)** Far UV-CD spectra of BSA in presence of increasing concentration (from 0.01 M to 0.1 Molar) MSG at 25°C, 25 mM phosphate buffer, and pH. 7.0. **(B)** The plot of mean residual ellipticity of BSA at 208 nm versus the concentration of MSG.

To know the effect of MSG on the secondary structure of protein BSA, far UV-CD was carried out, as depicted in Figure 7A. This experiment involves the titration of 5 μM BSA with the increasing concentration of MSG from 0.01 M to 0.1 M at the temperature of 25°C. Figure 7B shows the plot of mean residual ellipticity of BSA at 208 nm versus the concentration of MSG. Here, the experiment is performed at very low concentrations of MSG, since at 0.1 M MSG was involved in maximum denaturation of the secondary structure and the HT voltage value of the machine Jasco Spectropolarimeter crossed beyond 800. Beyond this HT voltage value, the CD signal becomes disproportionate, data cannot be valued, and it can damage the machine. Thus, to prevent this issue, an experiment was performed at low concentrations of MSG. From Figure 7A, it is clear that in the presence of MSG there is a decrease in the secondary structure of BSA. Even at the low concentration of MSG, there is complete perturbation and denaturation of the secondary structure of BSA. The peak present at 210 nm is completely vanished even at a very low concentration, 0.001 M of MSG. Upon addition of further concentrations which includes 0.02 to 0.1 M of MSG, there is formation of a new peak at 226 nm. This is a very interesting observation as far as the effect of MSG on the secondary structure analysis by Far-UV CD measurement is considered.

### Isothermal Titration Calorimetry Measurements

**FIGURE 8:**
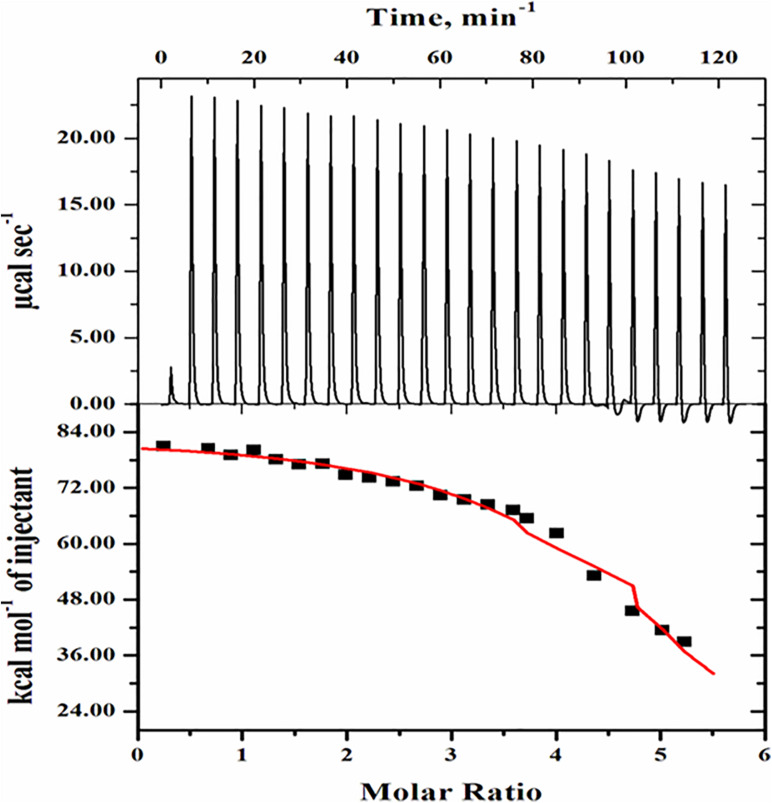
Isothermal calorimetric titration of BSA in presence of monosodium glutamate. The top side panel shows the raw data in the power versus time (heat per unit of time released after each injection of the ligand into the protein). The bottom side panel shows the raw data in the power standardization to the amount of injections (kcal mol^−1^) versus its molar ratios with the addition of consecutive injections of monosodium glutamate into the cell reaction containing BSA protein solution.

Figure 8 shows ITC measurement in which ligand MSG was titrated against the cell containing BSA. The concentration of BSA was 30 micromolars, and the ligand MSG was 900 micromolars. For determination of thermodynamic parameters, soft interaction, binding affinity, the determination of energetics of interaction of promoters and suppressors with the protein the pathway, and protein aggregation by quantifying the heat that is either liberated or consumed between MSG and BSA, ITC measurement was carried out.

The topmost side panel shows the raw data in power versus time (heat per unit of time liberated from every injection of the ligand with respect to the protein). This portion displays the data in 25 injections. The bottom side panel shows the raw data in the power standardization to the quantity of injections (kcal mol^–1^) against its molar ratios with the addition of consecutive injections of ligand into the cell reaction comprising the protein solution. The amount of heat released as a function of the mole ratio of ligand to protein is shown in the bottom side panel. Further, in the bottom side panel, dots denote the experimental data and the line correlates the best-fitting model holding the number of identical and independent binding sites. The Origin software installed in the VP-ITC Calorimeter was used to analyze routine data and their fitting in the form of isotherm displaying the summaries about heat changes in the interaction. Table 1 shows the various parameters, which include the association constant (*K*_*a*_), binding enthalpy (*ΔH*), and equilibrium constant (K_*d*_) accompanied with the ITC thermogram of BSA-MSG. Applying equation (2), (*ΔG*) free energy change was determined. The equilibrium dissociation constant was also evaluated (i.e., *K*_*d*_ = 1/*K*_*a*_) from the value of binding affinity as given in Table 1.

**TABLE 1:** Binding parameters of BSA with monosodium glutamate evaluated from ITC measurements at pH 7.0 and 25°C.

**Thermodynamic parameters (units)**	**N**	***K*_*a*_ (M^–1^)**	**Δ*H* (cal mol^–1^)**	**Δ*S* (cal mol^1^ deg^–1^)**	**Δ*G* (cal mol^–1^)**
Step 1	5.18 (± 0.079)	1.82 × 10^5^ (±3.31 × 10^5^)	8.328 × 10^4^ (± 1263)	303	−7014

## Discussion

Statistically, it has been estimated that the annual global demand for dietary MSG is near about 4 million metric tons (Markit, 2018). Asian countries are responsible for 88 percent of consumption, and consumption of China alone is 55 percent of the world’s consumption, as shown in Supplementary Figure 2A as the pie chart (Handbook, 2015). China is globally the largest exporter, giving 44 percent of MSG to the world. Major countries which serve as export destinations for the Chinese MSG are as shown in Supplementary Figure 2B as the pie chart (Plateform, 2014). In recent years, consumption of dietary MSG has been observed to be increasing throughout the globe as the most extensively used flavoring agent in order to enhance and improve the taste in foods. It is predicted to witness a significant growth of USD ∼ 6,200 million of Global MSG in 2022 (Future, 2020). Therefore, despite such a huge global consumption of MSG, still MSG is undoubtedly the controversial focal point as MSG consumption in both human and animal studies has been linked with various neurological and physiological complications (Fuchsberger, 2019). The degeneration of the nervous system by the progressive loss of structure or function especially in the neurons of the brain is called neurodegeneration; a heterogeneous group of diseases occurs due to the process of neurodegeneration, including Alzheimer’s diseases, Parkinson’s disease, Huntington’s disease, amyotrophic lateral sclerosis, multiple sclerosis, and Prion diseases, which are considered neurodegenerative diseases (Olney, 1960; Beas-Zarate et al., 1989; Samuels, 1999; Fraser, 2017; Shannon et al., 2017; Butnariu and Sarac, 2019; Fuchsberger, 2019). Supplementary Figure 3 illustrates the possible impacts of excessive dietary MSG consumption on human health. The neurodegenerative diseases are associated with protein misfolding and aggregation. The association of MSG consumption with neurodegenerative diseases represents an important medical challenge to human beings. There are no studies about the role of MSG with protein folding, unfolding, misfolding, and aggregation. Thus, this is the first study that primarily involves the investigation of aggregation behaviors of bovine serum albumin in the presence of MSG.

The results from the kinetic measurements of BSA in the presence of increasing concentrations of MSG at 60°C temperature revealed that MSG was involved in the aggregation of BSA. The change in kinetic parameters such as *A*_*max*_, *b*, *y*_*o*_, t½, (t_*lag*_), and Kapp min^–1^, associated with aggregation of BSA in the presence of MSG at varying concentrations from 0.1 to 4.5 M at temperature 60°C, is shown in Table 2. From this table, it is clear that *A*_*max*_ from 0.00289 to 0.2110 and *K*_*app*_ from 0.21 to 0.48 increase whereas *b* from 4.70 to 2.12, t½ from 34.0 to 5.54, and *t*_*lag*_ 24.6 to 1.43 decrease. On the other hand, there was further increase in the aggregation of BSA by MSG at 70°C. The change in kinetic parameters such as *A*_*max*_, b, *y*_*o*_, *t*½, t_*lag*_, and *K*_*app*_ min^–1^, associated with aggregation of BSA in the presence of MSG at varying concentrations from 0.1 to 4.3 M at temperature 70°C, is shown in Table 3. From this table, it is clear that *A*_*max*_ from 0.04 to 0.88 and *K*_*app*_ from 0.04 to 0.43 increase whereas b from 24.7 to 2.21, t½ from 51.26 to 4.11, and t_*lag*_ from 1.78 to 0.59 decrease.

**TABLE 2:** Change in kinetic parameters associated with aggregation of BSA in presence of monosodium glutamate at varying concentration from (0.1 to 4.5 M) at temperature 60°C.

**S.No.**	**Co-solvents**	**a**	***y*_*o*_**	**b**	**t ½**	**t_*lag*_ (min)**	***K*_*app*_ (min^–1^)**
1	No Additives	0.00289	0.0029	4.70	34.0	24.6	0.21
2	0.1 M	0.0089	1.0152	4.30	9.66	1.06	0.23
3	0.2 M	0.0252	3.705	4.05	6.43	1.67	0.25
4	0.4 M	0.0420	0.111	2.97	9.98	4.04	0.33
5	0.6 M	0.0517	0.071	2.94	8.96	3.08	0.34
6	0.8 M	0.0669	0.1255	2.37	7.46	2.54	0.42
7	0.9 M	0.101	0.053	2.18	5.80	0.60	0.38
8	1.0 M	0.129	0.181	2.67	8.28	0.78	0.37
9	2.0 M	0.137	0.356	2.67	6.61	0.85	0.37
10	2.5 M	0.177	0.791	2.49	5.56	0.99	0.40
11	3.0 M	0.184	0.968	2.11	5.54	1.32	0.47
12	4.0 M	0.196	0.947	2.11	5.52	1.33	0.47
13	4.5 M	0.2110	1.1053	2.12	5.54	1.43	0.48

**TABLE 3:** Change in kinetic parameters associated with aggregation of BSA in the presence of monosodium glutamate at varying concentrations from 0.1 to 4.5 M at temperature 70°C.

**S. no.**	**Co-solvents**	**a**	***y*_*o*_**	**b**	**t ½**	**t_*lag*_**	***K*_*app*_**
						**(min)**	**(min^–1^)**
1	No additives	0.04	0.0035	24.7	51.26	1.78	0.040
2	0.1 M	0.100	0.0086	16.92	86.01	54.00	0.06
3	0.5 M	0.15	0.0125	17.05	87.94	53.00	0.05
4	0.8 M	0.27	0.0169	19.57	87.57	48.32	0.05
5	1 M	0.45	–0.006	6.00	38.00	26.00	0.16
6	2 M	0.50	0.0176	5.67	32.87	23.39	0.17
7	2.5 M	0.62	0.0068	5.62	23.97	9.43	0.17
8	3 M	0.81	–0.0057	2.79	11.70	6.11	0.35
9	4 M	0.84	–0.0949	2.41	7.08	2.26	0.41
10	4.3 M	0.88	–0.2331	2.31	4.11	0.59	0.43

Furthermore, at temperature of 70°C, the aggregation kinetics of BSA in the presence of MSG was characterized by the presence of maximum turbidity. The pattern of MSG-induced aggregation of BSA occurs via a nucleation–polymerization mechanism and can be represented as the mathematical function having a characteristic of the sigmoid growth curve which entails a distinct lag or nucleation phase, tracked by a growth phase and subsequently by a saturation phase. In the nucleation phase, first the monomers of protein go through some modifications or conformational rearrangement and there are accumulation monomers which interact to form the nucleus of protein aggregate referred to as seeds or start aggregate or critical nucleus. The nucleation step is coupled with maximum activation energy or a transition state of maximum free energy in the pathway of aggregation and hence is thermodynamically unfavorable. There can be also the involvement of different protein intermediates such as partially denatured or mutated protein monomers and varied sizes of unstable oligomers. This step is reversible in nature and is the slowest or a rate-limiting step, which decides the rate of the whole aggregation kinetics. The start aggregates, which are small in size, can interact with the different cellular organelles and can turn toxic in nature. This interaction obstructs the functioning of proteasome and thus the aggregated protein cannot be degraded (Proctor et al., 2010). The occurrence of the lag phase comprehensively disturbs the speed of kinetics in protein aggregation and is frequently accompanied with increase in the concentration of nuclei during the course of the nucleation phase (Gelamo and Tabak, 2000; De et al., 2005; Librizzi and Rischel, 2005; Sharma et al., 2010b; Saha et al., 2016). At the temperature of 60°C, MSG-induced aggregation BSA is characterized by the absence of the lag phase; however, at the temperature of 70°C, MSG-induced aggregation BSA is outlined by means of the well-established lag phase. The lag time of the aggregation kinetics of 5 μM BSA was noticed to be 1.78, but in the presence of 0.1 molar MSG, the lag time of BSA aggregation was noticed to be increased abruptly about 54.00 per minute. Further increasing the concentration of MSG from 0.1 to 4.3 molar subsequently reduces the lag time from 54.00 to 0.59, as shown in Table 3. The reduction of the lag phase may be due to the addition of new seeds in the nucleation step of BSA aggregation by MSG. There are reports that suggest that addition of new seeds reduces the lag phase. The rate of aggregation kinetics can be accelerated and reduced by the addition of performed fibrils through a process called seeding. It can be either through the addition of new seeds of the same protein (homologous seeding or self-seeding) or through the addition of new seeds of heterologous seeding or cross-seeding of different proteins. Thus, MSG may be involved in the induction of homologous seeds, which in turn reduces the lag phase of aggregation. In the growth phase, from the existing critical nucleus, aggregates further develop promptly in their size into the different types of aggregates like amyloid or amorphous aggregates, which are found to be associated with neurodegeneration. This step is irreversible in nature, and the concentration of nuclei remains constant (Dobson, 2006; Morris et al., 2009; Dasgupta and Kishore, 2017). Eventually, all the proteins are being incorporated into aggregates with no additional attachment of fibrils and the depletion of monomers marks the saturation phase.

To know whether MSG has any inhibitory effect or not on the aggregation kinetics, the increasing concentration of MSG was titrated against CTAB-induced BSA aggregation. The results revealed that CTAB-induced BSA aggregation in the presence of MSG was near the aggregation of CTAB-induced BSA. The change in kinetic parameters such as *A*_*max*_, b, *y*_*o*_, *t*½, *t*_*lag*_, and *K*_*app*_ min^–1^, associated with CTAB-induced BSA in the presence of MSG at varying concentrations from 0.1 to 4.5 M at temperature of 70°C is shown in Table 4. From this table, it is clear that *A*_*max*_ from 0.00076 to 3.08 and *K*_*app*_ from 0.21 to 9.34 increases significantly whereas b from 4.70 to 0.107, t½ from 34.43 to 0.84, and t_*lag*_ from 25.03 to 0.62 decrease. Thus, MSG was again involved in the induction of homologous seeds, which in turn reduces the lag phase of both BSA aggregation as shown in Figure 2 and CTAB-induced BSA aggregation as shown in Figure 5 and Supplementary Figure 1B. Furthermore, the influence of temperature on the kinetics of MSG-induced BSA aggregation was carried out in the presence of a fixed concentration, 1 molar, of MSG at 60°C to 85°C temperatures. The change in kinetic parameters such as A_*max*_, b, *y*_*o*_, t½, t_*lag*_, and *K*_*app*_ min^–1^ was associated with BSA aggregation in the presence of a fixed concentration (1 molar) of MSG at variant temperatures starting from 60 to 85 as shown in Table 5. From this table, it is clear that *A*_*max*_ from 0.120 to 0.494 and *K*_*app*_ from 0.99 to 1.47 increase significantly whereas b from 1.01 to 0.68, *t*½ from 4.28 to 1.56 and (*t*_*lag*_) 2.28 to 0.20 decrease. Here the lag phase was not prominent and even changing the temperature from 60°C to 85°C is not involved in inducing the seeding phenomenon in the nucleation phase of aggregation. However, there is a substantial increase in BSA-MSG aggregation, with the increase in absorbance. The plot between lnt*_*lag*_* versus the reciprocal of temperature for MSG-induced aggregation of BSA at various temperatures depicts that this plot illustrates the nonlinear relationship with ln *t*_*lag*_ and there is a decrease in the lag time with the increase in temperature. All inset plots including *A*_*max*_ versus concentration at 60°C, *A*_*max*_ versus concentration at 70°C, and ln*t*_*ag*_ versus reciprocal of temperature show the significant nonlinear relationship in the turbidity measurements. Reports suggest that nonlinear relationships are associated with changes in the size of aggregates or changes in the morphology of aggregates in the turbidity measurements (Borgia et al., 2013). From 60 to 85°C temperatures, values of the rate constant acquired from time-dependent kinetic measurement were then used to evaluate the value of activation energy of the aggregation process as shown in Table 6. The results from the nonlinear fit of the plot of ln tlag vs. 1000/T associated with the nucleation phase of aggregation and the nonlinear fit of the plot of ln(*k*) vs. 1000/T was associated with nucleation growth of aggregation. Both plots depict a noticeable non-Arrhenius behavior when plotted over the full temperature range from 60 to 85°C. It has been reported that the protein aggregation which is promoted by temperature frequently shows the nonlinear behavior, and this type of aggregation is also referred to as non-Arrhenius protein aggregation (Wang and Roberts, 2013). Also, the report suggests that a change in temperature leads to a rate-limiting step of aggregation kinetics (Wang and Roberts, 2013), so the association of the non-Arrhenius behavior with the nucleation phase of MSG-BSA aggregation in the presence of temperature is well justified. Unfolding of protein is also accompanied with the increase in the activation energies (Lee and Timasheff, 1981; Sharma et al., 2010b), and here MSG was also observed with the increase in the activation energies.

**TABLE 4:** Change in kinetic parameters associated with CTAB-induced BSA aggregation in the presence of monosodium glutamate at varying concentrations from 0.1 to 4.5 M at temperature 70°C.

**S. no.**	**Co-solvents [BSA 40 μM + CTAB 1200 μM + MSG M]**	**a**	***y*_*o*_**	**b**	**t ½**	**t_*lag*_ (min)**	***K*_*app*_ (min^–1^)**
1	Control	0.00076	0.002	4.70	34.43	25.03	0.21
2	40 + 1200 + 2.5	3.15	1.40	0.21	0.30	0.37	4.7
2	40 + 0 + 2.5	0.297	0.018	19.57	87.46	48.32	0.05
3	40 + 1200 +2.5	0.629	0.006	7.018	23.79	9.76	0.14
4	40 + 1200 + 2.7	1.953	0.03	1.953	12.11	8.20	0.51
5	40 + 1200 + 3	2.32	0.05	0.442	3.47	2.59	2.27
6	40 + 1200 + 3.5	2.857	0.02	0.219	1.49	1.05	4.76
7	40 + 1200 + 4	2.91	0.005	0.131	1.33	1.06	7.63
8	40 + 1200 + 4.5	3.08	0.107	0.107	0.84	0.62	9.34

**TABLE 5:** Change in kinetic parameters associated with BSA aggregation in the presence of fixed concentration (1 molar) of monosodium glutamate at different temperatures ranging from 60 to 85°C.

**S. no.**	**Temperature (°C)**	**a**	***y*_*o*_**	**b**	**t ½**	**t_*lag*_ (min)**	***K*_*app*_ (min^–1^)**
1	60	0.120	0.015	1.5	5.80	2.28	0.67
2	63	0.195	0.023	1.31	4.56	1.94	0.76
3	65	0.228	0.013	1.13	4.12	1.86	0.88
4	70	0.248	0.012	1.00	3.93	1.92	1.00
5	75	0.265	0.007	0.57	3.14	1.99	1.75
6	78	0.353	0.010	0.59	3.17	1.99	1.69
7	80	0.406	0.010	0.59	3.17	1.99	1.69
8	81	0.431	0.013	0.59	2.17	0.99	1.69
9	82	0.490	0.023	0.48	1.44	0.48	2.08
10	84	0.488	0.023	0.55	1.69	0.59	1.81
11	85	0.494	0.020	0.73	1.56	0.20	1.47

**TABLE 6:** Calculation of activation energies into two segments from nonlinear behavior shown by slopes by plotting ln tlag vs. 1000/T and ln(*k*) vs. 1000/T.

**E_*a*_**	**Lag phase**	**Growth phase**
60 to 80°C	0.60 kcal/mol	9.90 kcal/mol
80 to 85°C	107.16 kcal/mol	4.44 kcal/mol

The effect of MSG on the reversibility of bovine serum albumin was also studied. Our results suggest that at 25°C, UV-visible spectroscopic studies of native BSA depict no scattering but in the presence of MSG a noticeable scattering was witnessed. However, after thermal denaturation of BSA in the presence of MSG at 80°C, the solution turns exceedingly turbid and aggregation was visible with the naked eyes. It is reported that the native protein is stabilized by a covalent bond (disulfide bonds) and noncovalent interactions or electrostatic interactions such as ionic bonds, hydrogen bonds, van der Waals forces, and hydrophobic interactions (Ankarcrona et al., 1995). The process by which protein loses its native, well-defined folded structure (i.e., secondary, tertiary, and quaternary structure) which is formed under physiological conditions to an unfolded state or biologically inactive state (in which primary structure is retained) under non-physiological conditions or under some external stress is known as denaturation (Tanford, 1968). There is change in the physical, chemical, and biological properties of native protein by virtue of denaturation (Dill and Shortle, 1991). In denaturation, covalent peptide bonds are not disrupted; however, there is disruption of covalent interactions between disulfide bridges between cysteine groups, noncovalent dipole–dipole interactions between polar amino acid side chains, Van der Waals interactions between nonpolar amino acid side chains, alpha-helices and beta-pleated sheets, and random coil configuration (Bazoti et al., 2005; Sionkowska, 2005). It may not involve complete unfolding of proteins, but there can be the presence of a folded structure in random conformation. It may provide an unfolded state to the protein without any noncovalent interactions responsible for the folded protein and denatured state to protein with some noncovalent interactions responsible for the folded protein stability. The changes occurring in one part of protein induces the unfolding of the other part; thus, denaturation is cooperative in nature (Wang and Spector, 2000). Cooling the thermally denatured BSA in the presence of MSG at a temperature of 25°C or overnight incubation at 25°C, in both circumstances, UV-visible studies suggest that all peaks of the BSA spectra were absent, totally disproportionate, and flattened. Reports suggest that lowering the temperature of the solution, removal of denaturants, and readjusting the pH to the native state are some important processes through which most proteins may be refolded to their native state. The refolded proteins may restore their biological functions. Native and denatured states are in equilibrium with each other. Upon removal of denaturant, protein refolds back to native conformation from denatured state, which is called renaturation or refolding, thus denaturation may be reversible (Roussel et al., 2013). The process by which unfolded (denatured) proteins are returned back to their native state partially or fully is known as renaturation (Rakic, 2012). Figure 6A clearly depicts that BSA forms aggregates in the presence of MSG and the aggregates are not reversible but are irreversible. Irreversible aggregates are the aggregates formed by misfolding of native monomers into stable or net-irreversible or higher-molecular-weight species which when formed cannot be dissociated without subjecting them to very higher concentrations of chemical denaturants, pressure, or temperature or by diluting the solution and is driven by hydrophobic interactions and hydrogen bonds (De Young et al., 1993; Tennent et al., 1995; Brummitt et al., 2012). The secondary and tertiary structures may be lost with the formation of irreversible aggregates. The concentrations of different aggregate species regulate the relative rates of the different processes. It has been reported that there is change in the viscosity of the solution on the formation of irreversible aggregates (Cromwell et al., 2006). The separation of irreversible aggregates can be also achieved by column chromatography (Wilson and Smith, 1959). The presence of irreversible aggregates cause degradation on the quality of products (Jiskoot et al., 2012).

In order to investigate the effect of MSG on the hydrodynamic size of BSA, a dynamic light scattering (DLS) experiment of BSA was performed in the absence and presence of MSG. DLS is a well-established technique that provides important structural information regarding biological macromolecules in solution especially to measure values of the hydrodynamic diameter, polydispersity, and existence of aggregates in the protein solution. Results depict that the value of the hydrodynamic diameter of BSA was observed to be about 8.3 nm (4.14 nm hydrodynamic radius) at pH 7.0, which is close to reported values. It has been reported that the value of the hydrodynamic diameter at pH 4.5 to 9.2 varies from 9.6 to 7.5 nm (Martinez-Landeira et al., 2002; Castelletto et al., 2007a,b). However, there was abrupt enhancement in the values of hydrodynamic diameter by the addition of MSG to the solution of BSA protein from 8.3 to 68. 33 nm (see Figure 6B). Thus, MSG causes the enhancement of the hydrodynamic diameter of BSA which could be due to unfolding of the BSA protein or due to the existence of aggregates in the solution. Also from the results of DLS measurements, structural deformation of protein by the MSG can be well justified.

The effect of MSG on the secondary structure of bovine serum albumin was studied from far-UV CD measurements. Far-UV CD ranging from 240 to 180 nm corresponds to the absorption of the peptide bond which is asymmetric in nature so molecules asymmetric in nature exhibit the phenomenon of CD. Far-UV CD provides information about the content of the secondary structure of protein which includes α-helix and β-sheet, turn, and random coil (Kelly and Price, 2000). The results shown in Figure 7A illustrate that a low concentration of MSG causes the substantial perturbation and denaturation of secondary of BSA. Moreover, even at the concentration of 0.001 M of MSG the peaks of BSA existing at 208 and 222 nm absolutely disappeared. The native spectrum of BSA illustrates strong negative signals at 208 and 222 nm and there was also the abrupt decrease in the value of mean residual ellipticity (MRE) or the negative signals of CD at 208 and 222 nm.

The plot of mean residual ellipticity of BSA at 208 nm versus the concentration of MSG shown in Figure 7B depicts the decrease in the negative ellipticity of protein from −22236 to 10880 in the presence of low concentrations of MSG. At concentrations from 0.02 to 0.1 M, MSG results in the disappearance of original peaks but results in the formation of a new peak at 226 nm, which is an attention-grabbing observation as far as the influence of MSG on the secondary structure analysis by far-UV CD measurement is considered. The formation of a new peak at 226 nm is an indication of aggregated or disordered proteins. These disordered proteins are unstructured or partially structured, rich in random coils, and or pre-molten globules (Clements et al., 1992), and also these proteins are deprived of an ordered secondary structure and three-dimensional structure referred to as intrinsically disordered proteins (IDPs) and is noticed by CD as “random coil,” “unordered,” or “disordered.” This observation can provide some evidence to diseases triggered by protein misfolding (Anand et al., 2011; Habchi et al., 2014; Knowles et al., 2014; Lopes et al., 2014; Li et al., 2015). Reports also suggest that from the far-UV CD measurement there is the presence of a secondary structure mainly with α-helix and random coil by titration of SDS with acid-induced denatured cyt c (Keiderling and Xu, 2002; Xu and Keiderling, 2004). Thus, from the far-UV CD measurements, our results suggest that a low concentration of MSG is involved in the unfolding of a secondary structure of protein with the disappearance of original peaks and the formation of a unique peak.

The protein folding under optimum physiological conditions in which unfolded proteins fold into a three-dimensional native state and functional state. These native proteins are stabilized by intermolecular interactions, which are thermodynamically favorable and regulated process (Fersht and Daggett, 2002; Daggett and Fersht, 2003; Baldwin, 2007; Nickson and Clarke, 2010). Figure 8 represents the ITC profile acquired after titration of BSA with MSG at pH 7.0 specifies with the positive heat pulse in the upper panel describing the binding of MSG with BSA. The interaction is endothermic in nature with a stoichiometry of n = 5.18 (± 0.079), *K*_*a*_ (M^–1^) = 1.82 × 10^5^ (± 2.6 x 10^4^), Δ*H* = {8.328 × 10^4^ ± 1263} cal mol^–1^, and Δ*S* = 303 cal mol^–1^ deg^–1^. From these binding parameters, using equation (2), change in Gibbs free energy was also calculated (Δ*G* = -7014 cal mol^–1^). Different binding and thermodynamic parameters associated between BSA-MSG interactions estimated from ITC measurements at pH 7.0 and 25°C are shown in Table 1. ITC thermogram data reveals the best-fitting with one site-binding model. The enthalpy change calculated is mostly positive which also suggests that the binding of interaction is endothermic in nature, entropy change (Δ*S*) calculated is positive, and Gibbs free energy change (Δ*G*) is largely negative which means the reaction is spontaneous in nature (Perozzo et al., 2004; Jia et al., 2014). There is noticeable heat of interaction or a specific heat pattern that mentions the binding affinity of MSG with BSA which is very much appreciable. Evaluation of properties of heat reactions including sign, magnitude, pattern, and shape on the thermogram is very much significant in determining the formation of different types of aggregates and has been accompanied with aggregation kinetics of proteins (Ikenoue et al., 2014). The ionic interaction, hydrogen bonding, hydrophobic interaction, and van der Walls forces have a vital role in binding affinity of protein with ligand (Sharma et al., 2010a; Ikenoue et al., 2014), and the endothermic heat of interaction between BSA and MSG indicates involvement of a hydrophobic interaction (Perozzo et al., 2004; Song et al., 2016; Patel and Bummer, 2017). A higher value of endothermic heat of interaction Δ*H* = {832.8 × 10^3^ (±1263 × 10^3^)} is suggestive of protein–MSG interaction or the formation of a protein–MSG complex. We speculate that this coupling of MSG with BSA causes aggregation in the protein (Ghosh et al., 2018). As there are reports suggesting that calcium causes the aggregation of α-synuclein with endothermic heat of interaction (Jain and Bhat, 2014), SDS-induced hydroxypropyl methylcellulose (HPMC) aggregation was also observed to be endothermic in nature (Patel and Bummer, 2017) and the polymer-induced surfactant aggregation is also coupled with endothermic heat of interaction (Loh et al., 2016).

## Conclusion

In conclusion of this work, we present the first report in support of the association of dietary MSG with protein misfolding and aggregation. This study demonstrates that MSG is involved in the promotion of protein aggregation via the mechanism of nucleation-dependent polymerization. CTAB-BSA aggregation was also promoted considerably in the presence of MSG. There is temperature dependence, non-Arrhenius nature, and endothermic heat of interaction revealed by MSG-induced BSA aggregation. MSG is involved in a marked increase in hydrodynamic diameter of the native structure which is the mark of unfolding and aggregation. Irreversible aggregate formation is caused by MSG which was also observed to be associated with the unfolding of the secondary structure of protein with the vanishing of native protein peaks and the formation of an exclusive peak, which is an important finding. This is the first study which justifies the role of MSG in protein misfolding and aggregation and provides the commencement for further investigations of the role of MSG in protein misfolding and aggregation, their exact mechanism, and their relationship with human physiological and neurodegenerative diseases.

## Author Contributions

IA performed all the experiments of biophysical and aggregation studies and prepared the first draft of the manuscript. ZP and SB assisted in the ITC experiment. AS, FA, MIH, MFA, AH, and AI designed the experiments, monitored the experimental work, and prepared the final draft of the manuscript. All authors contributed to the article and approved the submitted version.

## Conflict of Interest

The authors declare that the research was conducted in the absence of any commercial or financial relationships that could be construed as a potential conflict of interest.
