# Supplementary material for: Rationalizing the Role of Monosodium Glutamate in the Protein Aggregation Through Biophysical Approaches: Potential Impact on Neurodegeneration
Source: Front Neurosci. 2021 Mar 4;15:636454. doi: 10.3389/fnins.2021.636454 (PMC7969894; doi:10.3389/fnins.2021.636454)
Supplement: Supplementary file 1 [file Data_Sheet_1.DOCX]

Supplementary Material





**Supplementary Figure 1.**


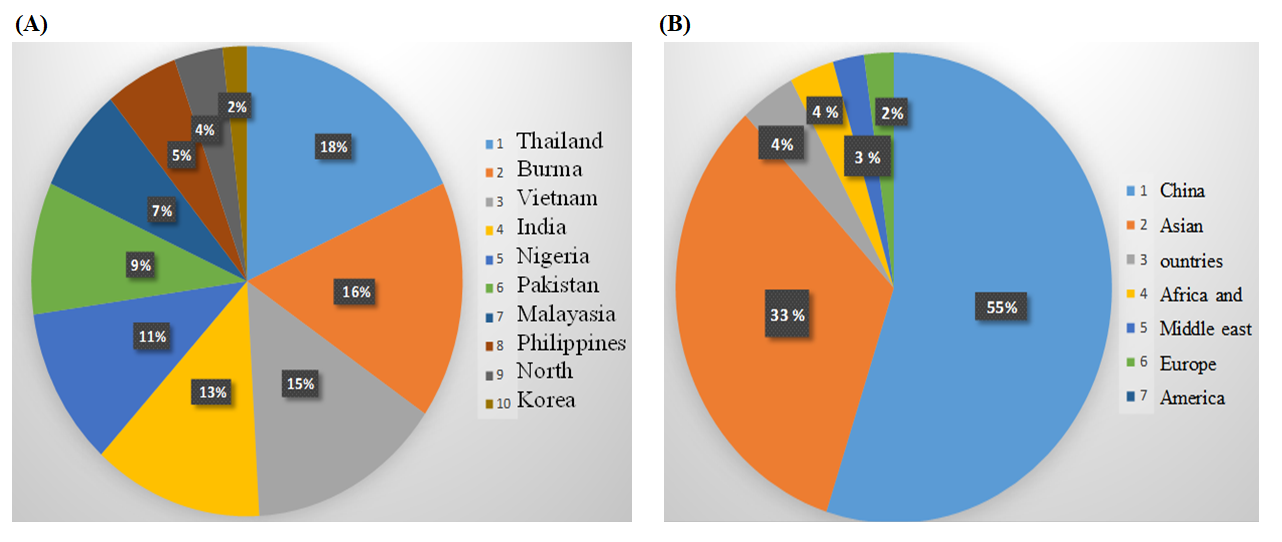


**Supplementary Figure 2.**


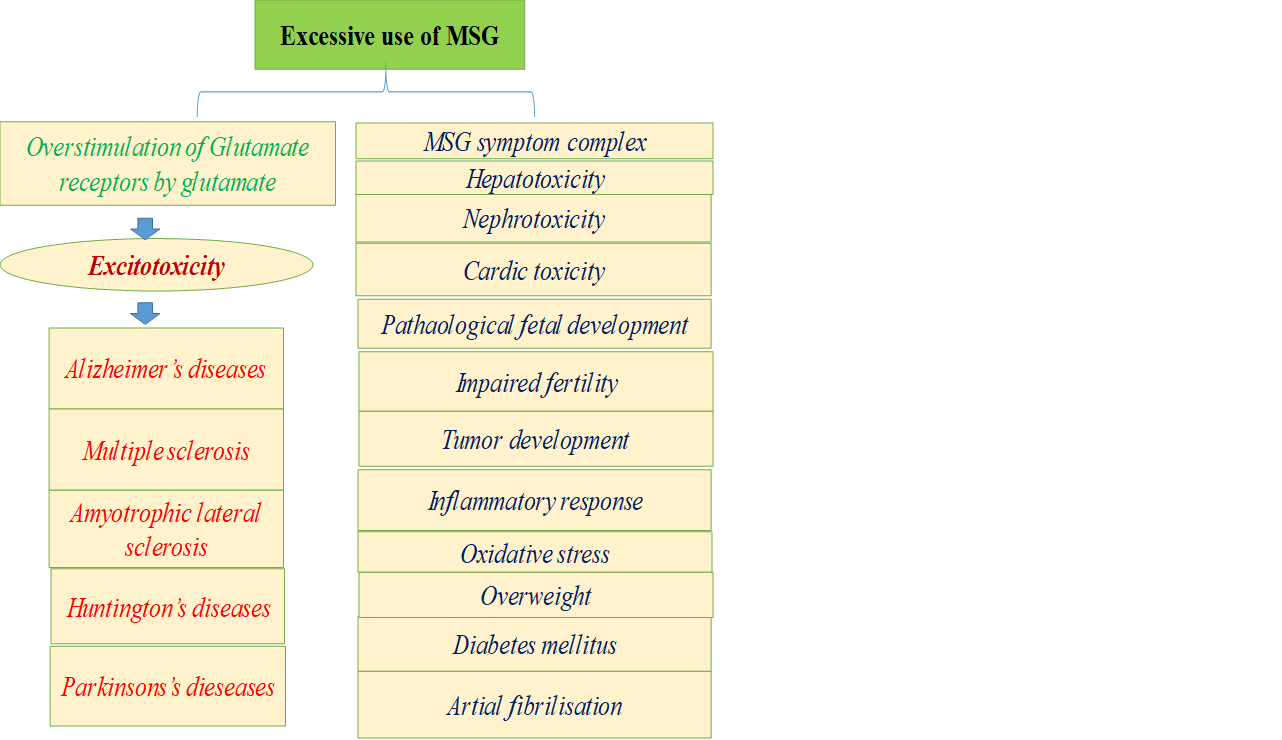


**Supplementary Figure 3.**

**Supplementary Figure Legends**

**Supplementary Figure 1.** Kinetic aggregation depicts both MSG and CTAB acts as the homologous seeds in the nucleation phase of BSA aggregation*.*

**Supplementary Figure 2. (A)** Depicting the percentage consumption of dietary MSG by Asian countries. **(B)** Depicting the major countries which serves as export destinations for the Chinese MSG.

**Supplementary Figure 3**. Depicting the possible impacts excessive of dietary monosodium glutamate on the human health.
